# A lysin to kill

**DOI:** 10.7554/eLife.16111

**Published:** 2016-04-27

**Authors:** Anthony O Gaca, Michael S Gilmore

**Affiliations:** 1 Department of Ophthalmology, Massachusetts Eye and Ear Infirmary, Harvard Medical School, Boston, United States; 1 Department of Ophthalmology, Massachusetts Eye and Ear Infirmary, Harvard Medical School, Boston, United Statesmichael_gilmore@meei.harvard.edu; 2 Department of Microbiology and Immunobiology, Harvard Medical School, Boston, United States; 2 Department of Microbiology and Immunobiology, Harvard Medical School, Boston, United States

**Keywords:** bacteriophage, membrane protein, endolysin, structure/function, Human, <i>S. pyogenes</i>

## Abstract

An enzyme produced by a bacteriophage can enter human cells and kill intracellular *Streptococcus pyogenes*.

**Related research article** Shen Y, Barros M, Vennemann T, Gallagher DT, Yin Y, Linden SB, Heselpoth RD, Spencer DJ, Donovan DM, Moult J, Fischetti VA, Heinrich F, Lösche M, Nelson DC. 2016. A bacteriophage endolysin that eliminates intracellular streptococci. *eLife* 5:e13152. doi: 10.7554/eLife.13152

**Image** An antimicrobial enzyme (green) co-localizes with, and ruptures, bacteria inside human cells

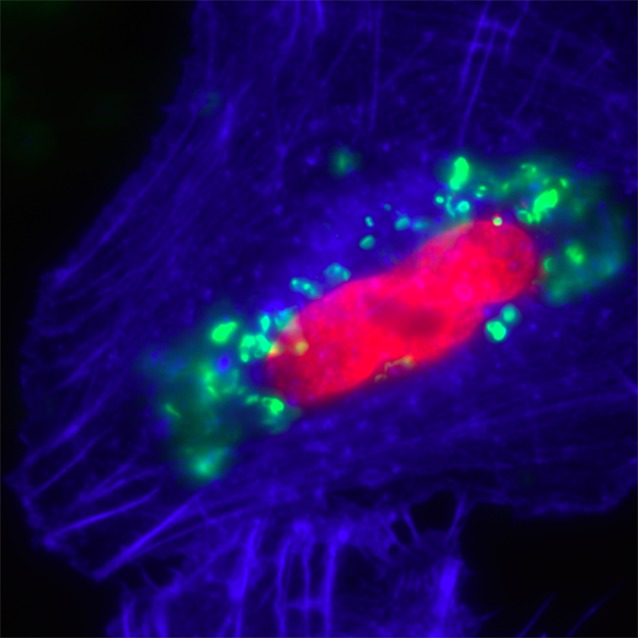

Being in the right place at the right time is a recipe for success for an antibacterial compound. *Streptococcus pyogenes* (often simply called *Spy*) is a bacterium that is responsible for a range of human infections, from the common “strep throat” to necrotizing fasciitis, the life-threatening condition also known as “flesh-eating disease” (Ralph and Carapetis, 2013). *Spy* infections can often return after treatment because the bacteria can invade human cells, which protect them from both our immune system and antibiotics (Osterlund et al., 1997). Now, in eLife, Daniel Nelson from the University of Maryland and co-workers – including Yang Shen (Maryland) and Marilia Barros (Carnegie Mellon University) as joint first authors – report the unexpected ability of an antimicrobial enzyme called PlyC to find and kill *Spy* that is hiding inside human cells (Shen et al., 2016).

PlyC is produced by a bacteriophage, a virus that infects bacterial cells. Like all viruses, bacteriophages commandeer their host’s replication machinery to produce copies of themselves. Some bacteriophages also carry genes for enzymes called endolysins or phage lysins that allow them to escape from the bacteria they have invaded by degrading the mesh-like cell wall that protects each bacterial cell.

The medical potential of bacteriophages and phage lysins was recognized shortly after they were discovered over a century ago. In fact, bacteriophage treatment was used for a range of illnesses in the early 20^th^ Century (Chanishvili, 2012), but it was quickly overshadowed by small-molecule antibiotics from the 1940s onwards. The widespread use of antibiotics in medicine and agriculture has, unfortunately and inevitably, led to the spread of antibiotic-resistant bacteria and a possible end to the “Antibiotic Era” (Harrison and Svec, 1998; Boucher et al., 2009). Also, more people are now aware that using broad-spectrum antimicrobial drugs can alter the microbial community in the gut, which in turn can impact on human health (Cotter et al., 2012).

Phage lysins possess a number of good antimicrobial traits. They are potent, fast-acting, specific to a narrow range of bacteria and relatively harmless to other types of cells. They can also kill bacteria that are dormant or not actively growing (Fischetti, 2010), and have been used to cure short-term *Spy* infections in mice (Gilmer et al., 2013). However, most had assumed that these enzymes were incapable of entering into host cells, and unlikely to be useful for treating chronic *Spy* infections in humans.

Shen, Barros and colleagues, who are based at various centers across the United States, attempted to overcome this assumed limitation by fusing phage lysins with fragments of proteins that help transport other molecules through cell membranes. Serendipitously, they discovered that PlyC is intrinsically active against intracellular *Spy*. PlyC is the only known phage lysin that is composed of two subunits. One of these subunits (PlyCA) is enzymatically active and degrades the bacterial cell wall. The other subunit (PlyCB) binds to the *Spy* cell surface and, as discovered by Shen, Barros et al., is needed to get the enzyme inside a human epithelial cell (Figure 1).

**Figure 1.**
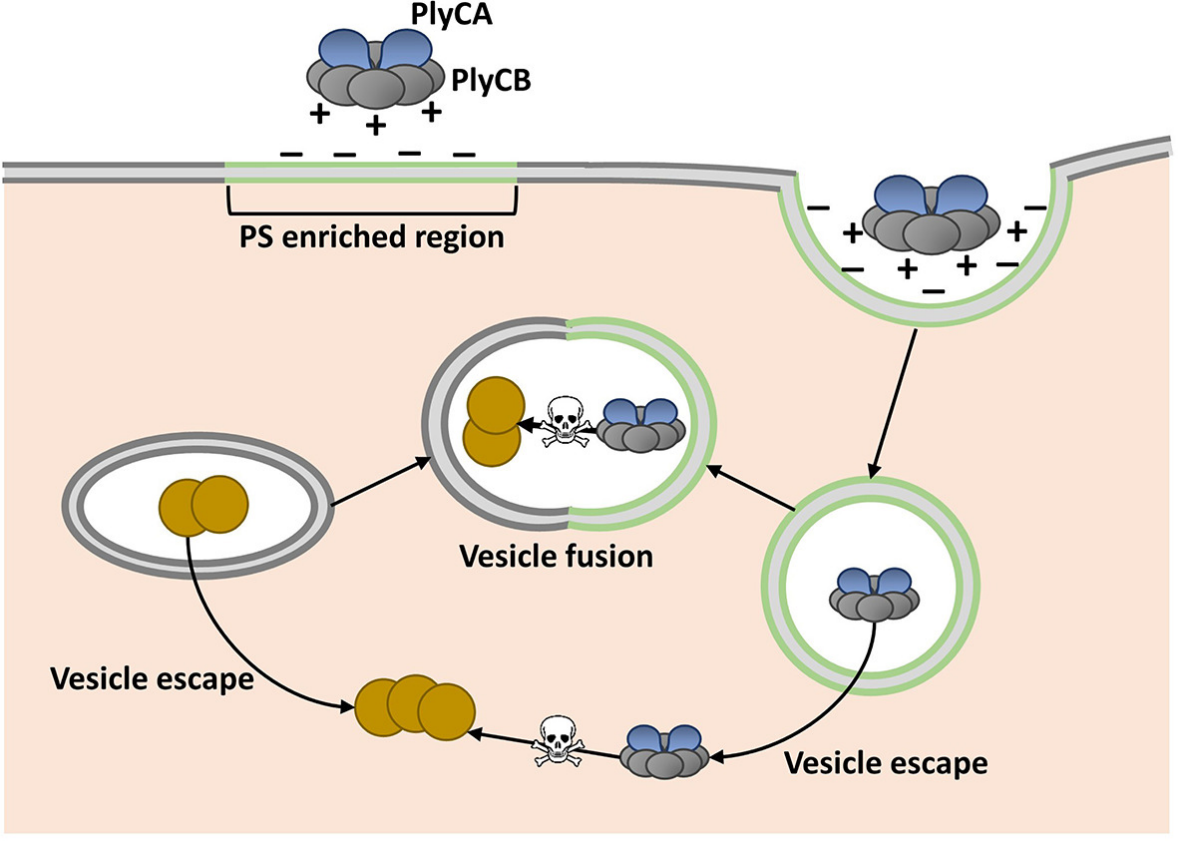
Model showing how the enzyme PlyC can enter human epithelial cells and kill intracellular *Streptococcus pyogenes (Spy*). PlyC is composed of two subunits. PlyCB forms an eight-part ring that normally binds to the *Spy* cell surface. PlyCA is the enzymatically active subunit that degrades the bacterial cell wall. The PlyCB surface is normally positively charged, and Shen, Barros et al. show that it interacts with phosphatidylserine (PS), which is negatively charged. They propose that PlyCB binds to lipid rafts (shown in green) that are enriched in PS; this causes the membrane to fold around PlyC, which ultimately enters the cell inside a vesicle. These vesicles may fuse with bacteria-containing vesicles, giving PlyC access to intracellular *Spy* (depicted as brown circles). Alternatively, PlyC may escape the vesicle and interact with and kill *Spy* that is free in the host cell.

The PlyCB subunit was known to have a positively charged surface (McGowan et al., 2012), and so Shen, Barros et al. hypothesized that it interacts with negatively charged components of the cell membrane. Indeed, they then went on to discover that PlyCB binds to a negatively charged molecule called phosphatidylserine that is common in eukaryotic cell membranes. Their results also suggested that PlyCB can only penetrate membranes that contain at least 30% phosphatidylserine.

Regions of the cell membrane called lipid rafts have high levels of phosphatidylserine, and are involved in the uptake of numerous molecules from the cell’s exterior via a process called endocytosis (Pike, 2009). This suggested that PlyC might enter cells via an endocytic mechanism, and Shen, Barros et al. provided further support for this idea by showing that PlyCB co-localizes with another protein that binds to lipid rafts. Based on these data and some molecular modeling, they proposed that PlyCB binds to phosphatidylserine, causes the membrane to fold around it, and ultimately enters the cell via endocytosis (Figure 1).

These new findings demonstrate the potential of phage lysins, and possibly bacteriophage-based therapy, to treat challenging bacterial infections. Specifically, they also highlight the potential of PlyC to reduce or even prevent infection with *Spy*, which remains a major health concern in low- to middle-income countries and disproportionately targets children (Ralph and Carapetis, 2013). Further work is needed to confirm if PlyC enters cells via lipid raft-mediated endocytosis, and to determine how PlyC ultimately meets the intracellular *Spy* in order to kill it. However, insights provided by Shen, Barros et al. open up the possibility of engineering new endolysins based on PlyCB to target other disease-causing microbes that evade the immune system by hiding in an infected person’s cells.
